# Machine learning to determine optimal conditions for controlling the size of elastin-based particles

**DOI:** 10.1038/s41598-021-85601-y

**Published:** 2021-03-18

**Authors:** Jared S. Cobb, Alexandra Engel, Maria A. Seale, Amol V. Janorkar

**Affiliations:** 1 Biomedical Materials Science, School of Dentistry, University of Mississippi Medical Center, 2500 North State St. D528, Jackson, MS 39216 USA; 2 Information Technology Laboratory, US Army Engineer Research and Development Center, 3909 Halls Ferry Rd, Vicksburg, MS 39180 USA

**Keywords:** Biomedical materials, Biomaterials

## Abstract

This paper evaluates the aggregation behavior of a potential drug and gene delivery system that combines branched polyethyleneimine (PEI), a positively-charged polyelectrolyte, and elastin-like polypeptide (ELP), a recombinant polymer that exhibits lower critical solution temperature (LCST). The LCST behavior of ELP has been extensively studied, but there are no quantitative ways to control the size of aggregates formed after the phase transition. The aggregate size cannot be maintained when the temperature is lowered below the LCST, unless the system exhibits hysteresis and forms irreversible aggregates. This study shows that conjugation of ELP with PEI preserves the aggregation behavior that occurs above the LCST and achieves precise aggregate radii when the solution conditions of pH (3, 7, 10), polymer concentration (0.1, 0.15, 0.3 mg/mL), and salt concentration (none, 0.2, 1 M) are carefully controlled. K-means cluster analyses showed that salt concentration was the most critical factor controlling the hydrodynamic radius and LCST. Conjugating ELP to PEI allowed crosslinking the aggregates and achieved stable particles that maintained their size below LCST, even after removal of the harsh (high salt or pH) conditions used to create them. Taken together, the ability to control aggregate sizes and use of crosslinking to maintain stability holds excellent potential for use in biological delivery systems.

## Introduction

Protein and biopolymer-based drug and gene carriers transport their cargo through many different methods such as chemical conjugation, non-covalent association by electrostatic interactions, or by physically packaging the therapeutic product through a conformational change^1^. An optimal delivery system requires low cytotoxicity, efficiency in cellular and nuclear uptake, targeting specificity, and a modifiable platform to enable adaptation for different physiological environments and dosage control^1^. Current analytical and biochemical technologies allow the design of drug and gene delivery systems on nano and micromolecular scales, and recent innovative approaches have led to the development of hybrid systems that combine a therapeutic agent with molecules designed to carry that therapeutic agent to a specific location within the body^1,2^. Another important factor for delivery systems is the ability to vary the size of the drug carrier depending on the drug type and delivery method. For example, smaller carriers (≤ 200 nm) achieve better intracellular delivery. Small particles also offer a large surface area, which can increase the rate of drug release^3^. Larger carriers (≥ 1 μm) are useful in vivo when a slow release of the drug is required, as is the case with slowly released hormones and for the delivery of insulin^4^. Large particles are also useful when a size-limiting barrier is used to hold the carriers in a particular area; for example, having the particles reside in the lungs or liver as opposed to the kidneys^3,4^. Another use for larger carriers is in dry powder formulations for inhalers. A particle radius of 1 to 5 μm is desired because it gives the particles enough mass that their movement is not solely governed by Brownian motion, and the particles can settle on a target surface. This size is also large enough that the particles stay localized in the lungs^5^.

Elastin-Like Polypeptides (ELP) belong to a family of genetically engineered biopolymers that have become popular for drug and gene delivery^6–23^. ELP has a repeating amino acid sequence of Val-Pro-Gly-X-Gly (VPGXG) based on the sequence of mammalian elastin, where the guest residue, X, can be any amino acid except proline. ELP has a characteristic lower critical solution temperature (LCST) above which it undergoes a reversible inverse phase transition, changing from a disordered hydrated polymer to a more structurally ordered coacervate^7–9^. This LCST can be raised or lowered by modifying the ELP composition or by changing the polymer concentration and ionic strength present in solution^10–13^. Such potential for ELP’s LCST modification and ELP’s ability to exhibit the inverse phase transition behavior even when it is conjugated to other proteins have led to its applications in drug delivery, protein purification, and tissue engineering^14–18^. Conjugation of functional or therapeutic peptides to ELP, either by chemical reaction with guest residues or ELP terminal ends, has also enabled the creation of ELP-based fusion proteins. These molecules combine the precisely regulated and thermo-induced assembly of ELP with other functional constructs that can be packaged into nanoparticles for biological applications^19–24^.

Lone PEI is an effective synthetic trans-membrane carrier but is known to be both cytotoxic and hemolytic^25,26^. PEI, when combined with a large biocompatible molecule, is less cytotoxic^27–31^. Previously, we have shown that ELP-PEI copolymers exhibit cell biocompatibility when used as cell culture coatings^32,33^. Turner et al. conjugated an 800 g/mol PEI to the carboxylic acid end of ELP and coated it onto a cell culture plate to encourage three-dimensional spheroid formation by murine adipocytes^34^. In this culture system, the PEI created a positively charged surface that prevented the cells from fully adhering, and forced them to adopt a three-dimensional configuration. It was shown that the cells showed greater differentiation compared to the traditional two-dimensional monolayer culture^34^.

While several studies have reported the LCST behavior of ELP, there are no quantitative ways to control the size of aggregates formed after the phase transition to a size greater than 200 nm^35–40^. Moreover, as the phase transition behavior of the ELP is reversible for many variants, the aggregates formed above the LCST dissolve away once the solution temperature is lowered below the LCST. Addressing this critical gap in the way ELP aggregate will significantly enhance their utility as drug and gene delivery carriers. To this end, we present a method wherein manipulating three solution conditions/environmental factors, namely the pH, polymer concentration, and salt concentration, can achieve precise aggregate radii for neat ELP. We further demonstrate that this control can be retained for neat ELP mixed with a small fraction of ELP conjugated to two different molecular weights (800 and 10,000 g/mol) of PEI. ELP/ELP-PEI copolymer mixtures also show a similar LCST behavior as the neat ELP. More importantly, we show that the PEI can serve as a crosslinking site to maintain the size of the aggregates of ELP/ELP-PEI copolymer mixtures even after changing the variables/environmental factors, including lowering the solution temperature below the LCST, removal of the added salt, and adjusting the solution to neutral pH.

## Materials and methods

### ELP expression

ELP (MW = 17,000 g/mol; [VPGVG]_40_) was produced from genetically modified *E. coli* BLR (DE3) bacteria (*Novagen EMD*), and purification was accomplished through an inverse phase transition procedure involving cycles of solubilization at 4 °C and precipitation at 40 °C. Our lab has published a comprehensive account of this procedure^32,34^.

### ELP-PEI copolymer synthesis

ELP (30 mg) was reacted to branched PEI (MW = 800 g/mol from Sigma-Aldrich St. Louis, MO, and MW = 10,000 g/mol from Polysciences Warrington, PA) by first reacting the carboxylic acid terminal end group of ELP to N-hydroxysuccinimide (10:1 molar ratio of NHS: ELP) using 1-ethyl-3-(3-dimethyl-aminopropyl) carbodiimide (EDC) as a catalyst (10:1 molar ratio of EDC: ELP) in 3 mL MES buffer at a pH of 6.2 for 15 min. PEI (10:1 molar ratio PEI: ELP) was added to a separate container and dissolved in 2 mL MES buffer and subsequently titrated to a pH of 6.5. The PEI solution was cooled to 4 °C, and the ELP-NHS solution was added dropwise while stirring the PEI solution. This reaction was allowed to proceed overnight at 4 °C. Removal of unreacted PEI was achieved by inverse phase transition purification and dialysis (8000 g/mol cutoff, 1 L of DI water), of which a detailed account has been previously published^32,33^. The resulting copolymers are referred to as ELP-PEI800 and ELP-PEI10K. An average of 27 mg of mixtures of unreacted ELP and ELP-PEI copolymer was recovered for both reactions. The average reaction conversion for the ELP to PEI800 reaction was 29%, and for the ELP to PEI10K reaction was 9.2% as determined by the O-phthalaldehyde (OPA) assay. The assay was performed according to the manufacturer’s instructions. In brief, the ELP/ELP-PEI copolymer mixtures (dissolved in DI water) were treated with OPA (Thermo Scientific, Waltham, MA), and the fluorescence emission at 455 nm was measured with a Biotek FLx 800 fluorescence plate reader (Winooski, VT) and compared to a generated standard curve.

### Sample preparation

The 29% and 9.2% conversions for the ELP-PEI800 and ELP-PEI10K reactions meant that we had mixtures containing 29 mol% ELP-PEI800/71 mol% neat ELP and 9.2 mol% ELP-PEI10K/90.2 mol% neat ELP after the reactions, respectively. Neat ELP was then added to the ELP/ELP-PEI800 mixture to bring the molar ratio to 15:85 ELP-PEI800: neat ELP as this ratio was previously shown to exhibit no cytotoxicity when used as a cell culture coating^33,34^. Neat ELP was then added to the ELP/ELP-PEI10K mixture to bring the molar ratio to 1.3:98.7 ELP-PEI10K: ELP. This molar ratio was chosen to maintain the same number of amine groups (crosslinking sites) in the ELP/ELP-PEI10K mixture as those present in the ELP/ELP-PEI800 mixture. Keeping the number of amine groups the same allowed us to investigate the effect PEI molecular weight had on aggregate formation and crosslinking.

Aqueous solutions of ELP/ELP-PEI copolymers were prepared by altering the overall polymer concentration (0.1, 0.17, 0.3 mg/mL in deionized water), solution pH (3, 7, 10), and salt concentration (0, 0.2, 1 M NaCl). Neat ELP was tested at all solution variations as a control for comparison. The polymer concentrations were chosen to maximize the success of forming particles that could be later used in biological applications. As an example, a representation of the solution composition would be [pH = 3, 0.1 mg/mL, 1 M NaCl].

Samples were diluted with Milli-Q deionized water to the proper polymer concentration, NaCl was added, and the pH was altered with the dropwise addition of 1 M NaOH or 1 M HCl using an Accumet pH meter. Samples with added salt were placed back in a 4 °C refrigerator for at least 15 min to maintain a temperature below 20 °C. NaCl can drastically lower the LCST of ELP, and solutions with salt were refrigerated to prevent phase transition at room temperature.

### Dynamic light scattering (DLS)

**Figure 1:**
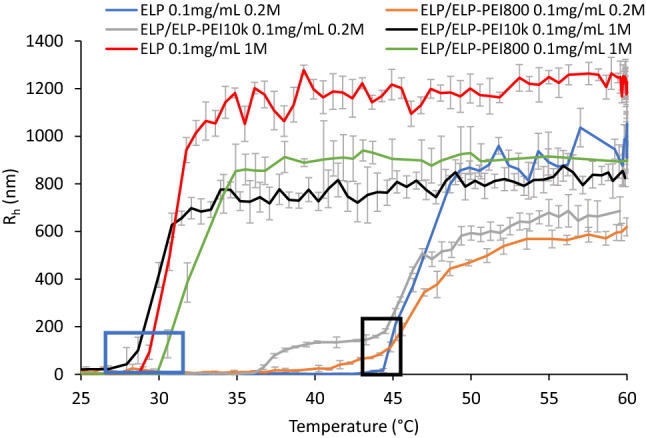
DLS results demonstrate single and bimodal curves can be obtained by varying the salt concentration. The second transition for the bimodal curve for the ELP-PEI10K (grey) occurs at the same point (black box) as neat ELP (blue) and ELP/ELP-PEI800 (orange), indicating that either the ELP-PEI10K transitions earlier than neat ELP or it has more than one ELP molecule conjugated to the PEI10K. The bimodality of the ELP/ELP-PEI800 (orange) and the ELP/ELP-PEI10K (grey) curves disappears with an increase in NaCl concentration to 1 M (blue box). All DLS curves were obtained at pH = 7.

A Dynapro Nanostar DLS instrument (*Wyatt Tech*) was used to determine aggregation sizes of the polymers over a temperature range of 20–60 °C with a ramp rate of 1 °C/min (n = 6) and a sample volume of 25 µL. Dynamics software version 7.1.9 was used to capture and analyze the data. Maximum hydrodynamic radii (R_h_) were calculated by averaging the mean R_h_ values above the LCST. The LCSTs represent the mean temperature at which the polymer begins to transition, and the DLS curve begins to rise (Fig. 1). The 95% confidence intervals for T_t_ are negligible due to the 0.1 °C temperature accuracy of the DLS.

### Crosslinking of ELP-PEI copolymers

The ELP/ELP-PEI copolymer solutions were heated above their respective LCST in an oven. The LCSTs of the four compositions tested ranged from 22 to 37 °C. Glutaraldehyde was then added (10:1 molar ratio of glutaraldehyde: ELP) and allowed to react with the ELP/ELP-PEI copolymer aggregates in the oven for 15 min. The samples underwent the inverse phase transition purification procedure as described above to remove the unreacted glutaraldehyde and the harsh (high salt or pH) conditions used to create the aggregates. To ensure the crosslinking was successful, and to determine the particle sizes of the crosslinked ELP/ELP-PEI copolymers, the samples were cooled to 4 °C, and DLS was performed at a static temperature of 20 °C, both of which are below the LCST of the non-crosslinked aggregates.

### K-means cluster analysis

K-means was performed in Python (v. 3.7.4) using Scikit-learn (v. 0.21.3) with the following parameters: n_clusters = 3, init = k-means++ , n_init = 10, max_iter = 300, precompute_distances = auto. Data manipulation was performed using Pandas (v. 0.25.1) and Numpy (v. 1.16.5). The number of clusters was determined using the silhouette score in Scikit-learn. The number of clusters with the largest silhouette score was chosen for the n_clusters parameter in k-means (S1).

### Statistical analyses

Maximum radii and LCST results are reported as mean ± 95% confidence interval. Statistical analyses were performed with ANOVA followed by the Tukey post hoc test. Results with p ≤ 0.05 were deemed significantly different.

## Results

### LCST behavior of neat ELP and ELP/ELP-PEI copolymer mixtures

**Figure 2:**
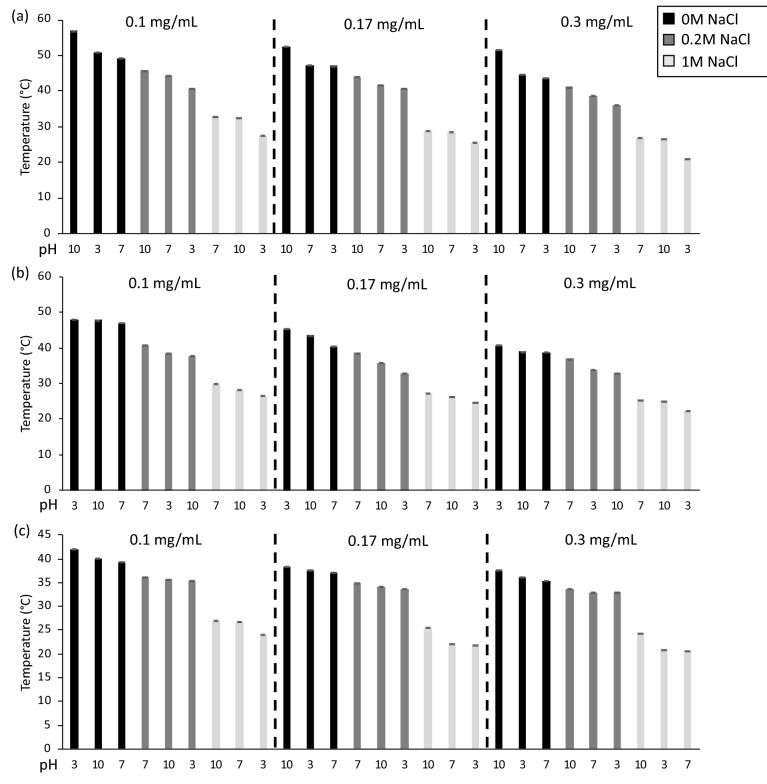
LCST of aqueous solution samples sorted from highest to lowest with respect to the concentration of (**a**) ELP, (**b**) ELP/ELP-PEI800, and (**c**) ELP/ELP-PEI10K as determined by dynamic light scattering. Statistical significance between samples can be found in Supplementary Figure S1.

Unaltered neat ELP (MW = 17,000 g/mol) was tested first to serve as a control against which we could compare the behavior of ELP/ELP-PEI copolymer aggregates. Chilkoti and coworkers have extensively reported on the LCST properties of ELP^11–13,15^. The current experiment serves as a new perspective on how the solution environment (polymer concentration, pH, and salt concentration) alters the LCST (Fig. 2a)^11–13,15^. For neat ELP, at pH = 3, an increase in polymer concentration and salt concentration decreases its LCST (Fig. 2a). In pH = 3 solution at 0.1 mg/mL, the ELP has an LCST of 50.8 °C which drops to 47.0 °C at an increased polymer concentration of 0.17 mg/mL (p < 0.05). With an increase of salt from 0 M to 0.2 M and polymer concentration held at 0.1 mg/mL, the LCST drops even further from 50.8 to 40.7 °C (p < 0.05). The same trends are seen for pH = 7 and 10. If both polymer concentration and salt concentration are held constant, an increase in pH causes a slight increase in LCST of neat ELP in most solution combinations (p < 0.05), indicating an effect of inherent pK_a_ of the only terminal carboxylic acid group present in the ELP. These results were expected based on existing literature^11,12^.

For ELP/ELP-PEI800, in pH = 3 solution, an increase in polymer concentration, as well as an increase in salt concentration, decreases LCST (Fig. 2b; p < 0.05). In pH = 3 solution at 0.1 mg/mL, ELP/ELP-PEI800 has an LCST of 48.0 °C which drops to 44.6 °C with an increased polymer concentration of 0.17 mg/mL. When concentration is held at 0.1 mg/mL, and NaCl is increased from 0 to 0.2 M, the LCST drops from 48.0 to 38.5 °C. The same trends are seen for NaCl addition at pH = 7 and 10. The downward shift in LCST caused by increasing polymer concentration is also seen at pH = 7 and 10. A change in pH has no significant impact on the LCST of ELP/ELP-PEI800 if salt and polymer concentration are held constant (p > 0.05).

As with neat ELP and ELP/ELP-PEI800, an increase in polymer concentration and salt concentration decreases the LCST of ELP/ELP-PEI10K (Fig. 2c; p < 0.05). In pH = 3 solution at 0.1 mg/mL concentration, ELP/ELP-PEI10K polymer mixture has an LCST of 42.1 °C which drops to 37.7 °C with an increase in polymer concentration to 0.17 mg/mL. If the concentration is held at 0.1 mg/mL, and NaCl is added at 0.2 M, the LCST further drops from 42.1 to 35.2 °C. Similar to ELP/ELP-PEI800, but in contrast to ELP, an increase in pH has no significant impact on LCST (p > 0.05).

### Aggregate sizes for neat ELP and ELP/ELP-PEI copolymer mixtures

**Figure 3:**
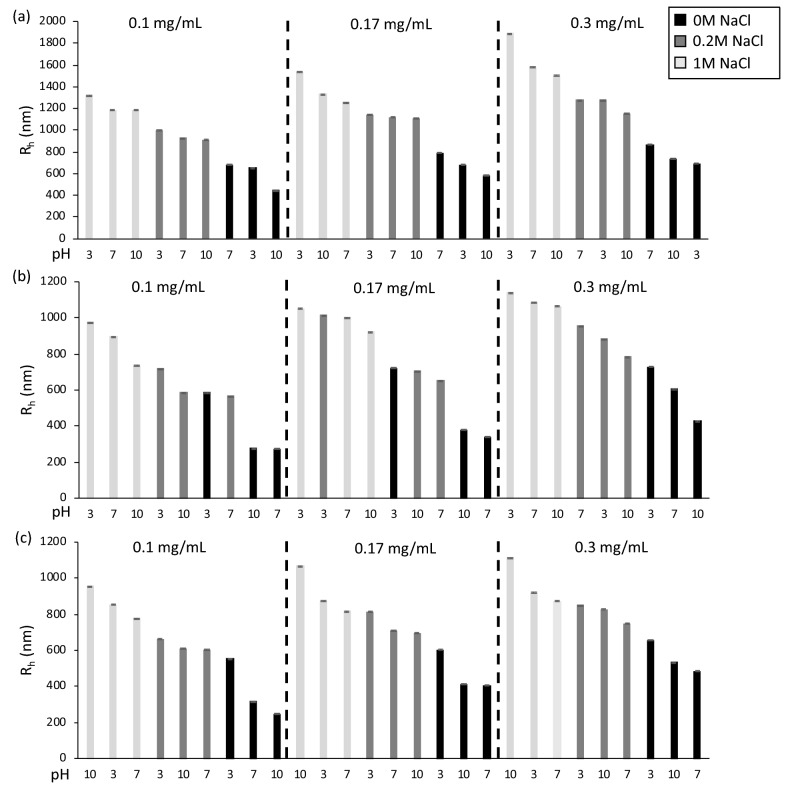
Dynamic light scattering results of aqueous solution samples for the R_h,_ sorted from largest to smallest with respect to polymer concentration of (**a**) ELP, (**b**) ELP/ELP-PEI800, and (**c**) ELP/ELP-PEI10K. Statistical significance between samples can be found in Supplementary Figure S2.

For neat ELP, changing the solution environment (polymer concentration, pH, and salt concentration) achieved aggregate sizes (hydrodynamic radii, R_h_) from 447 to 1894 nm (Fig. 3a). An increase in ELP concentration has a significant impact on mean R_h_ at 0.3 mg/mL (1223 ± 110 nm) compared to 0.1 mg/mL (925 ± 78 nm) and 0.17 mg/mL (1064 ± 86 nm). Salt concentration has a large impact on R_h_. For example, in pH = 3 solution at 0.1 mg/mL concentration, the R_h_ nearly doubles from 654 ± 12 nm in water (0 M NaCl) to 1002 ± 132 nm in 0.2 M NaCl (p < 0.05). Increasing the salt concentration to 1 M NaCl further increases the maximum R_h_ to 1324 ± 121 nm (p < 0.05). The same trends are seen for pH = 7 and 10. Alteration in solution pH, if both polymer concentration and salt concentration are held constant, has no significant impact on neat ELP aggregate radii (p > 0.05), which is the opposite of how pH affected the T_t_.

For ELP/ELP-PEI800, changing the solution environment achieved smaller aggregate sizes from 276 to 1141 nm (Fig. 3b) compared to those for neat ELP (Fig. 3a). In pH = 3 solution, increasing salt from 0 to 1 M increases the radius of ELP/ELP-PEI800 aggregates (Fig. 3b). The same trend is seen for increasing polymer concentration in pH = 3 solutions, where the radius increased with an increase in polymer concentration from 0.1 to 0.3 mg/mL. For pH = 7 and 10, independent increases in both polymer concentration and salt concentration yield consistently larger aggregate radii. With concentration held constant, increasing the solution pH from 3 to 7 or from 3 to 10 decreases R_h_ in 0 M and 0.2 M NaCl solutions.

For ELP/ELP-PEI10K, changing the solution environment achieved a similar range of aggregate sizes (Fig. 3c) to those for the ELP/ELP-PEI800 (Fig. 3b). In pH = 3 solution at all polymer concentrations, increasing salt concentration from 0 to 1 M NaCl increases the radius of the ELP/ELP-PEI10K aggregates. In pH = 7 and 10 solutions, increasing salt concentration from 0 M to 0.2 M and then to 1 M significantly increases the R_h_ of ELP/ELP-PEI10K aggregates (p < 0.05) (Fig. 3c). In pH = 7 and 10 solutions, an increase in polymer concentration increases R_h_ irrespective of salt concentration. However, this effect is not as pronounced as exhibited by the ELP/ELP-PEI800 solutions. In solutions with 0 M NaCl at all polymer concentrations, increasing the pH from 3 to 7 decreases R_h_, but a further increase to pH 10 has no additional impact on R_h_.

### Crosslinking maintains ELP particle stability and allows the removal of salt

**Figure 4:**
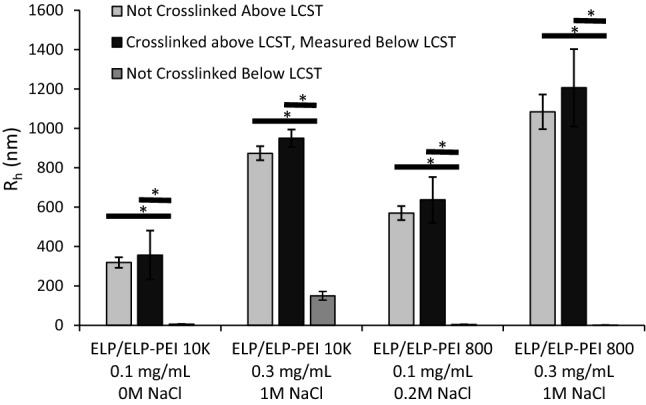
When heated above the LCST, the ELP/ELP-PEI polymers form aggregates that remain present in solution so long as the temperature is maintained (light grey bars). Upon crosslinking, the aggregates remain stable below their LCST at 20 °C (black bars), while the aggregates of the non-crosslinked polymers dissolve in the solution below their LCST at 20 °C (dark grey bars).

The light gray bars in Fig. 4 show the R_h_ measurements of several ELP/ELP-PEI polymer samples above the LCST. When the temperature is lowered to 20 °C, which is below the LCST of these polymers, the aggregates fall apart and dissolve in solution (Fig. 4 dark gray bars). Alternatively, these ELP/ELP-PEI polymer samples were heated above their LCST, crosslinked by glutaraldehyde, and re-suspended in DI water. The R_h_ measurements of the crosslinked particles (Fig. 4 black bars) were then taken at pH = 7 and a static temperature of 20 °C. As shown in Fig. 4, the crosslinking of polymers after aggregate formation maintains the aggregate radius below their LCST even after removal of the solution conditions (salt, pH) that were used to create the aggregates. For example, ELP/ELP-PEI800 at 0.3 mg/mL in a solution containing 1 M NaCl has R_h_ of about 1084 nm (light grey bar), which is similar to the R_h_ of the crosslinked ELP/ELP-PEI800 aggregates suspended in DI water (black bar).

### K-means cluster analysis

**Figure 5:**
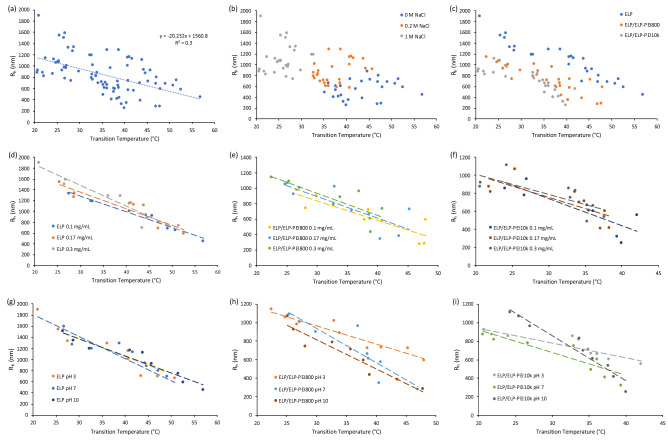
(**a**) A weak relationship was seen between R_h_ and T_t_ with an R^2^ of 0.3. (**b**) K-means cluster analysis was used to elucidate the primary driver of the relationship between R_h_ and T_t_. The three groups indicated by K-means cluster analysis to be important for determining R_h_ versus T_t_ are based on the concentration of salt. (**c**) Separating the R_h_ versus T_t_ graph by polymer type shows that increasing the molecular weight of PEI attached to ELP decreased the R_h_ and T_t_ simultaneously. Graphs for (**d**) ELP, (**e**) ELP/ELP-PEI800, and (**f**) ELP/ELP-PEI10K demonstrate that increasing polymer concentration decreases T_t_, but has a small influence on R_h_. (**g**) ELP exhibits minimal effects from a change in pH. Coupling ELP to PEI imposes a pH sensitivity that allows for a control over R_h_ for (**h**) ELP/ELP-PEI800 and (**i**) ELP/ELP-PEI10K.

Preliminary data exploration showed that there was a weak correlation between R_h_ and T_t_ (R^2^ = 0.3) (Fig. 5a). To further explore this relationship, K-means cluster analysis was used as a fast, non-biased way to construct an initial logical framework with which to begin to elucidate the underlying drivers between the relationship of R_h_ and T_t_. K-means is an unsupervised machine learning algorithm that finds related data by minimizing the distance between a randomly placed point and the surrounding data^41^. A more detailed explanation on how K-means establishes clusters can be found elsewhere^42^. The K-means analysis identified three clusters of data points corresponding to the three salt concentrations. We determined that these clusters correctly separated 90.1% of the data points based on the three salt concentrations, indicating that the salt concentration is the primary driver of the relationship between R_h_ and T_t_. The incorrectly classified data points in the three clusters were manually reassigned into the three respective salt concentrations (Fig. 5b). Figure 5b shows that by increasing salt concentration, the transition temperature is decreased for all three polymers, and simultaneously the R_h_ is increased. Separating the R_h_ vs. T_t_ graph by polymer type shows that, by increasing the molecular weight of the PEI attached to the ELP, the transition temperature and maximum R_h_ are simultaneously decreased (Fig. 5c).

It can be seen that salt concentration and polymer type have the most discernable influence on T_t_ and R_h_ (Fig. 5b,c), while pH and polymer concentration have less influence over T_t_ and R_h_ that is more difficult to isolate due to numerous competing factors (Fig. 5d–i). Still, qualitative trends can be seen using linear trend lines to discern the influence pH and polymer concentration have on the two outcomes (Fig. 5d–i). The length of the trend lines can be used to demonstrate the range of T_t_ values while the spread between the trendlines indicates the effect each parameter has on R_h_. A small spread between the trendlines indicates that polymer concentration has a small influence on the final R_h_ of the three polymers (Fig. 5d–f). Figure 5g shows minimal spread between the trendlines for ELP, indicating that pH has almost no influence on the R_h_. However, when PEI is coupled to the ELP the spread between the trendlines increases, demonstrating imposed pH sensitivity from the PEI (Fig. 5h–i).

## Discussion

### Effect of solution parameters on LCST

The LCST behavior of ELP in solution can be modulated by polymer concentration, NaCl content, and pH^10–13^. An increase in the concentration of ELP decreases the LCST by increased hydrophobic interactions, and does so quantitatively as a function of the natural logarithm of the ELP concentration^11^. We observed similar behavior in neat ELP (Fig. 2a). Both ELP/ELP-PEI copolymer mixtures also displayed lower LCST with higher polymer concentrations (Fig. 2b,c).

Cho et al. investigated the effect of the Hofmeister series ions on the LCST of ELP and found that NaCl exhibited a linear salting-out behavior, decreasing the LCST as a function of molar NaCl concentration. The Na^+^ and Cl^−^ ions cause a destabilization of the water molecules’ hydrophobic hydration of the polymer structure^12^. The same depression of LCST by NaCl was observed for neat ELP and both ELP/ELP-PEI copolymer solutions used in our study (Fig. 2). For the ELP-PEI copolymers, this effect is likely a combination of the disruption of hydrophobic hydration and the neutralization that occurs when Cl^−^ anions interact with protonated amines of PEI, which further encourages hydrophobic folding (Figs. 1 and 5).

Altering solution pH can be used as an effective method for tuning LCST when the ELP structure has ionizable residues at the guest position^10^. Our copolymer design differs in that the charged aspect of the copolymer is attached as a terminal controlling end and is not integral to the ELP molecule. For the [VPGVG]_40_ polymer, where all X = Valine, pH was not expected to significantly influence the LCST behavior of neat ELP, as seen in Fig. 2a. In the absence of salt, increasing pH had no effect on the LCST’s of ELP/ELP-PEI800 and ELP/ELP-PEI10K (Fig. 2b,c). Trabbic-Carlson et al. found that encoding a hydrophobic, neutral target protein to an ELP terminal end will depress the LCST of the reacted protein^43^. Similarly, when ELP-PEI copolymers are placed in water, without the anion interference of salt, an increase in pH deprotonates the PEI blocks, and the now more neutral non-transitioning blocks of PEI are forced together at a lower temperature by hydrophobic interactions between both the PEI and ELP blocks of the copolymer^5^.

The molecular weight of the PEI block progressively impacts the LCST (Fig. 2) as neat ELP solutions have higher LCSTs than the ELP/ELP-PEI800 solutions, which in turn have higher LCSTs than the ELP/ELP-PEI10K solutions under similar solution environment (polymer concentration, pH, and salt concentration) (Figs. 1 and 5). MacKay et al. found that adding more positive hydrophilic amino acids in the guest residue positions of the ELP raises the LCST, allowing better water solvation and increasing the temperature necessary to make transitioning energetically advantageous^10^. Therefore, in pH = 3 solution, one would assume that the ELP-PEI800 and ELP-PEI10K would have a higher LCST than the uncharged neat ELP because the positive charges on the PEI block would be expected to help maintain hydration at higher temperatures. Surprisingly, we found the opposite. One major difference between our study and that of MacKay et al. is that the hydrophilic groups are localized to one side of the molecule in ELP-PEI as opposed to being distributed evenly throughout the ELP. This could help the hydrophobic ELP blocks of the ELP-PEI more easily arrange to form an inner core of the aggregate surrounded by the solvated PEI blocks. Because of the highly hydrophilic nature of the protonated amine groups of the PEI, such a core–shell aggregate can form more favorably, which is at a lower temperature than the aggregate formation of neat ELP. On average, ELP/ELP-PEI10K appears to have an LCST 3.3 °C lower than that of ELP/ELP-PEI800, and ELP/ELP-PEI800 appears to have an LCST 4.1 °C lower than neat ELP. Alternatively, as shown by Weeks et al., the ELP-PEI10K may have more than one ELP molecule conjugated to the PEI10K, which transitions at a lower temperature by virtue of its higher molecular weight^44^.

### Effect of solution parameters on R_h_

The pH had no statistically significant effect on the R_h_ of neat ELP. This was expected as the ELP variant used in this study contains no ionizable groups. McKay et al. have previously noted this behavior and developed an ELP that contained evenly spaced ionizable residues. However, they performed optical density measurements which do not measure R_h_^10^. The aggregate radii of neat ELP were only affected by NaCl concentration and polymer concentration (Fig. 3a). Increasing salt and polymer concentration has been known to affect the R_h_. For example, Ghoorchian et al. found that by incrementally increasing the salt concentration from 5 to 60 mM, they could systematically increase the R_h_ of a three-armed star ELP from 13 to 78 nm^45^. The mechanism associated with the increase in R_h_ with increased salt concentration can be attributed to the same phenomena that depresses LCST. ELP is inherently hydrophobic, but remains thermodynamically stable at colder temperatures. As the temperature is increased, the salt decreases the thermodynamic stability of the ELP, and forces the hydrophobic sections to interact in order to decrease ELP’s contact with the surrounding water^12^. Non-miscible substances decrease their surface area with a solute, ultimately forming smaller particles. Salt is known to increase the surface tension of water and increase the amount of interaction between protein molecules. This increase in surface tension from the salt likely allows the insoluble ELP to form larger aggregates than in water alone. ELP polymers that form smaller R_h_’s (< 200 nm) than what is found in this study, and are coupled to other hydrophilic moieties, are thought to form micelles^35–40^. The hydrophobic sections occupy the core of the micelle where they can minimize the interaction with water, while the hydrophilic section remains soluble. Tuning the size ratio of hydrophobic to hydrophilic sections determines the micelles stability in water and the nm size of the micelle. The aggregates formed in our study are larger than 200 nm which indicates that they are true aggregates and not micelles. This means that the ELP and PEI sections are randomly dispersed within the aggregated structure. Conjugation of a PEI block to the non-ionizable ELP allows the R_h_ to be incrementally controlled by polymer concentration, salt concentration, and pH (Fig. 3b,c). ELP/ELP-PEI solutions at pH = 3 show the largest mean R_h_ at 870 ± 51 nm for ELP/ELP-PEI800 and 755 ± 37 nm for ELP/ELP-PEI10K versus 655 ± 66 nm and 638 ± 53 at pH 7 for the two polymers respectively (p < 0.05). This suggests that at pH = 3, there may be electrostatic repulsion of the entirely protonated PEI block driving the larger R_h_^46^.

As with LCST, an increase in pH to 10 decreases the R_h_ in the 0 M and 0.2 M NaCl solutions of ELP/ELP-PEI800 and ELP/ELP-PEI10K. As salt is added, it neutralizes the charges on the PEI blocks, and ionic disruption of solvation becomes the dominating factor in controlling the aggregate radius. ELP/ELP-PEI800 and ELP/ELP-PEI10K show statistically similar trends in regard to salt concentration (p > 0.05), with pH becoming a less important modulator of R_h_ at 1 M NaCl. Note that both ELP-PEI polymers were adjusted to have an equivalent number of amine groups, thus giving them an equal ability to be affected by salt concentration. Polymer concentration showed consistent trends for all three polymers with an increase in concentration resulting in a larger R_h_.

### K-means cluster analysis

To out knowledge, this research is the first time the K-means cluster analysis has been used to find patterns in polymer particle data. K-means is a simple but powerful algorithm that was able to quickly elucidate the relationship between salt concentration and T_t_ and R_h_. It should be noted that traditional subjective means of data exploration, as noted in Fig. 5, can also be used to find these relationships; however, K-means allows for a fast, un-biased way to begin breaking down complex data into understandable groups. A weak correlation was shown for T_t_ and R_h_ (Fig. 5a). The relationship between R_h_ and T_t_ was explored using K-means cluster analysis, which revealed the primary driver for both outcomes to be salt concentration followed by polymer type (Fig. 5b,c). In water, the effects from polymer concentration and pH were more pronounced with T_t_ varying over a 22 °C range and the R_h_ varying over a 621 nm range (Fig. 5b). The T_t_ range decreases from 22 to 13 °C, but the R_h_ range increases from 621 to 714 nm for 0.2 M samples (Fig. 5b). This indicates that salt concentration becomes the primary driver of T_t_, while a combination of salt and polymer type begins to influence the R_h_ with minor contributions from polymer concentration and pH (Fig. 5b). This trend continues for 1 M NaCl, with a decrease in T_t_ driven by salt, and an increase in R_h_ attributed to salt and polymer type with small variability driven by polymer concentration and pH (Fig. 5b). ELP and ELP/ELP-PEI800 are shown to have an inverse linear relationship between T_t_ and R_h,_ which means that as either R_h_ or T_t_ is increased, the other is decreased (Fig. 5d,e). ELP/ELP-PEI10K shows linearity for 0 M and 0.2 M but deviates at 1 M NaCl to lower R_h_ values (Fig. 5f). This indicates that the effect salt concentration has on ELP/ELP-PEI10K with respect to R_h_ decreases from 0.2 to 1 M compared to ELP and ELP/ELP-PEI800.

### Phase transitions and R_h_

As mentioned in the methods section, some of the polymer solutions exhibited multimodal phase transitions. ELP/ELP-PEI800 solutions almost exclusively exhibited a single-phase transition, whereas ELP/ELP-PEI10K exhibited bimodal transitions, except in the presence of high salt concentrations (Fig. 1). We suspect that the aggregates formed by the ELP/ELP-PEI copolymer solutions are a mixture of ELP-PEI copolymers and the neat ELP, as in any given solution, the fraction of ELP-PEI800 and ELP-PEI10K is only 15% and 1.3% respectively, with the rest of the polymers in the solution being neat ELP. We posit that there could be a hierarchical formation of early structures where ELP-PEI copolymers transition first and provide a thermodynamically favorable substrate that the neat ELP surrounding them can easily fold around. For the bimodal transitions of ELP/ELP-PEI10K, we surmise that when the R_h_ is reached for aggregates containing both ELP-PEI10K and neat ELP, the remaining ELP stay suspended in solution and transition later at a higher temperature almost equivalent to the LCST of neat ELP tested at those same conditions (Fig. 1). In contrast, the ELP-PEI800 exhibits a single-phase transition in all but three of the twenty-seven solution conditions tested. This is likely because the larger 17,000 g/mol ELP blocks in ELP-PEI800 and the neighboring neat ELP molecules can negotiate around the smaller non-transitioning 800 g/mol PEI blocks to continue to condense together.

Overall, the ELP/ELP-PEI solutions formed smaller aggregates than neat ELP in any given solution condition. Even though the ELP-PEI copolymers have a larger molecular weight and a smaller mole fraction in solution than the ELP, the electrostatic repulsion of the small portion of PEI copolymers makes it more difficult for the transitioning ELP blocks of ELP-PEI and the neat ELP polymers to coalesce. As previously stated, as pH increases, there is less protonation of the amines of PEI, and the ELP-PEI copolymers become a more neutral hydrophobic molecule that should behave similarly to neat ELP. At pH = 10, both ELP/ELP-PEI800 and ELP/ELP-PEI10K aggregate radii are closer in size to the R_h_ of neat ELP under the same solution conditions.

**Figure 6:**
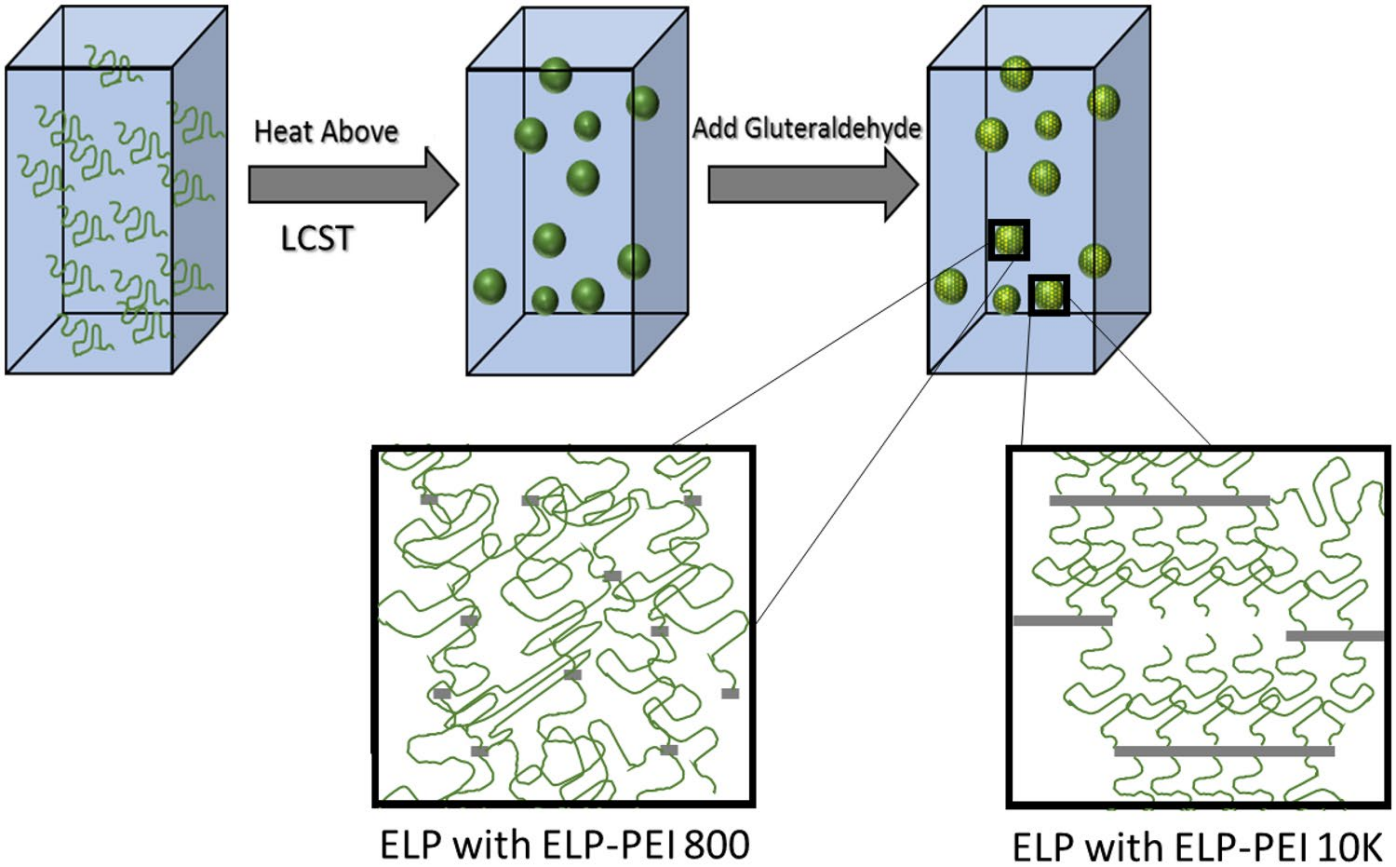
ELP/ELP-PEI800 and ELP/ELP-PEI10K systematically form nano-and micro-aggregates above LCST when the solution conditions of salt concentration, polymer concentration, and pH are carefully controlled. The PEI block provides the ability to crosslink the copolymers and to achieve particles that remain stable below LCST even after removal of the harsh (high salt or pH) conditions used to create them.

In this study, we have shown that ELP/ELP-PEI800 systematically forms nano-aggregates with a radius of 276 nm to microaggregates with a radius of 1141 nm with a range of LCST from 22 to 48 °C (Figs. 2b and 3b). These aggregates were achieved using the same chemistry of copolymer synthesis, but with careful manipulation of the solution environment. The same is true for ELP/ELP-PEI10K, where we can form nano-aggregates with a radius from 250 nm to microaggregates with a radius of 1114 nm with a range of an LCST from 20 to 42 °C (Figs. 2c and 3c). The PEI block also provides the ability to crosslink the copolymers and achieve a stable particle radius after formation in harsh environments (Fig. 4). These observations are schematically represented in Fig. 6.

In the future, ELP/ELP-PEI could serve as a modifiable platform that combines ELP’s capability for thermally induced self-aggregation and biocompatibility with the transfection efficiency and control of particle radius conferred by adding a block of PEI. ELP-PEI copolymers thus have potential as a biotherapeutic delivery agent whose dosage can be controlled by crosslinking the particles at the desired nano- or macromolecular size. This study serves as an initial investigation of the copolymers’ functionality, and more complex ELP-PEI copolymers can be created using the same chemistry. For this study, we chose the ELP with a repeat unit of VPGVG, but moving forward, alteration of the guest residue of ELP could provide further particle size and LCST customization of ELP-PEI copolymers. Similarly, chain length (molecular weight) is another quantifiable determinant of ELP behavior and may be an avenue for further modification. There are likely more complexities to the particle formation of the ELP-PEI copolymers to be explored, including isolation of pure copolymers (compared to the ELP/ELP-PEI mixtures used in this study) and an investigation of the early micelle structures formed in different environments before the aggregation takes place. This study provides the first step in understanding how the ELP-based copolymers behave in solution and shows their potential as functional biopolymers in an increasingly popular field of nanomolecular applications in biology and medicine.

## Conclusions

The LCST behavior of ELP has been extensively studied and can be controlled by many factors, including polymer concentration, Hofmeister cosolvents, pH, and molecular weight, but there are no quantitative ways to control the R_h_ of the polymer aggregates they form after the transition. Adding a PEI block to the terminal end of ELP allows the aggregate radius as well as LCST to be controlled by changing any combination of polymer concentration, salt concentration, and pH. The molecular weight of the added PEI block does not have a significant impact on radii between the two ELP-PEI copolymers in any given solution condition using the weight percentages chosen for this study. The molecular weight does, however, determine how responsive the copolymer will be to the small changes in solution conditions and hence its ability to be tailored for specific applications. Conjugation of an 800 g/mol PEI block is enough to significantly impact the behavior of ELP and provide control over aggregate size that does not exist for neat ELP. Overall, we have shown that salt concentration has a higher impact on polymer behavior than the polymer concentration for both ELP and ELP/ELP-PEI. This could mean that salt can be used for coarse tuning to get within a window of LCST and aggregate radius, while polymer concentration and pH can be used as modulators for fine-tuning of the copolymer properties.

## Supplementary Information

Supplementary Information 1.

Supplementary Information 2.

## References

[1] Kowalczyk T, Hnatuszko-Konka K, Gerszberg A, Kononowicz AK (2014). Elastin-like polypeptides as a promising family of genetically-engineered protein based polymers. World J. Microbiol. Biotechnol..

[2] Smits FCM, Buddingh BC, van Eldijk MB, van Hest JCM (2015). Elastin-like polypeptide based nanoparticles: Design rationale toward nanomedicine. Macromol. Biosci..

[3] Blanco E, Shen H, Ferrari M (2015). Principles of nanoparticle design for overcoming biological barriers to drug delivery. Nat. Biotechnol..

[4] Hoshyar N, Gray S, Han H, Bao G (2016). The effect of nanoparticle size on in vivo pharmacokinetics and cellular interaction. Nanomedicine.

[5] Telko M, Hickey A (2005). Dry powder inhaler formulation. Respir. Care.

[6] Rodríguez-Cabello JC, Arias FJ, Rodrigo MA, Girotti A (2016). Elastin-like polypeptides in drug delivery. Adv. Drug Deliv. Rev..

[7] Urry DW (1988). Entropic elastic processes in protein mechanisms. I. Elastic structure due to an inverse temperature transition and elasticity due to internal chain dynamics. J. Protein Chem..

[8] Urry DW (1992). Free energy transduction in polypeptides and proteins based on inverse temperature transitions. Prog. Biophys. Mol. Biol..

[9] Urry DW (1997). Physical chemistry of biological free energy transduction as demonstrated by elastic protein-based polymers. J. Phys. Chem. B.

[10] Mackay JA, Callahan DJ, Fitzgerald KN, Chilkoti A (2010). Quantitative model of the phase behavior of recombinant pH-responsive elastin-like polypeptides. Biomacromol.

[11] Meyer DE, Chilkoti A (2004). Quantification of the effects of chain length and concentration on the thermal behavior of elastin-like polypeptides. Biomacromol.

[12] Cho Y, Zhang Y, Christensen T, Sagle LB, Chilkoti A, Cremer PS (2008). Effects of Hofmeister anions on the phase transition temperature of elastin-like polypeptides. J. Phys. Chem. B..

[13] McDaniel JR, Radford DC, Chilkoti A (2013). A unified model for de novo design of elastin-like polypeptides with tunable inverse transition temperatures. Biomacromol.

[14] Amruthwar SS, Janorkar AV (2013). In vitro evaluation of elastin-like polypeptide-collagen composite scaffold for bone tissue engineering. Dent. Mater..

[15] Christensen T, Hassouneh W, Trabbic-Carlson K, Chilkoti A (2013). Predicting transition temperatures of elastin-like polypeptide fusion proteins. Biomacromol.

[16] Bessa PC, Machado R, Nürnberger S, Dopler D, Banerjee A, Cunha AM, Rodríguez-Cabello JC, Redl H, van Griensven M, Reis RL, Casal M (2010). Thermoresponsive self-assembled elastin-based nanoparticles for delivery of BMPs. J. Control Release..

[17] Kwon SH, Cho H (2012). Non-chromatographic method for the hepatitis B virus X protein using elastin-like polypeptide fusion protein. Osong Public Health Res. Perspect..

[18] Yang K, Su Y, Li J, Sun J, Yang Y (2012). Expression and purification of the antimicrobial peptide cecropin AD by fusion with cationic elastin-like polypeptides. Protein Expr. Purif..

[19] Massodi I, Thomas E, Raucher D (2009). Application of thermally responsive elastin-like polypeptide fused to a lactoferrin-derived peptide for treatment of pancreatic cancer. Molecules.

[20] Dreher MR, Raucher D, Balu N, Michael Colvin O, Ludeman SM, Chilkoti A (2003). Evaluation of an elastin-like polypeptide-doxorubicin conjugate for cancer therapy. J. Control Release..

[21] Bidwell GL, Raucher D (2010). Cell penetrating elastin-like polypeptides for therapeutic peptide delivery. Adv. Drug Deliv. Rev..

[22] Hassouneh W, Zhulina EB, Chilkoti A, Rubinstein M (2015). Elastin-like polypeptide diblock copolymers self-assemble into weak micelles. Macromolecules.

[23] Dai M, Frezzo JA, Sharma E, Chen R, Singh N, Yuvienco C, Caglar E, Xiao S, Saxena A, Montclare JK (2016). Engineered protein polymer-gold nanoparticle hybrid materials for small molecule delivery. J. Nanomed. Nanotechnol..

[24] Bidwell GL, Davis AN, Fokt I, Priebe W, Raucher D (2007). A thermally targeted elastin-like polypeptide-doxorubicin conjugate overcomes drug resistance. Investig. New Drugs..

[25] Boussif F, Lezoualc’h MA, Zanta MD, Mergny D, Scherman B, Demeneix JPB (1995). A versatile vector for gene and oligonucleotide transfer into cells in culture and in vivo: Polyethyleneimine. Proc. Natl. Acad. Sci..

[26] Godbey WT, Wu KK, Mikos AG (1999). Poly(ethylenimine) and its role in gene delivery. J. Control Release..

[27] Moghimi SM, Symonds P, Murray JC, Hunter AC, Debska G, Szewczyk A (2005). A two-stage poly(ethylenimine)-mediated cytotoxicity: Implications for gene transfer/therapy. Mol. Ther..

[28] Brus C, Petersen H, Aigner A, Czubayko F, Kissel T (2004). Physicochemical and biological characterization of polyethyleneimine-graft-poly(ethylene glycol) block copolymers as a delivery system for oligonucleotides and ribozymes. Bioconjug. Chem..

[29] Godbey WT, Wu KK, Mikos AG (2001). Poly(ethylenimine)-mediated gene delivery affects endothelial cell function and viability. Biomaterials.

[30] Tian H, Xiong W, Wei J, Wang Y, Chen X, Jing X, Zhu Q (2007). Gene transfection of hyperbranched PEI grafted by hydrophobic amino acid segment PBLG. Biomaterials.

[31] Teo PY, Yang C, Hedrick JL, Engler AC, Coady DJ, Ghaem-Maghami S, George AJ, Yang YY (2013). Hydrophobic modification of low molecular weight polyethyleneimine for improved gene transfection. Biomaterials.

[32] Janorkar AV, Rajagopalan P, Yarmush ML, Megeed Z (2008). The use of elastin-like polypeptide-polyelectrolyte complexes to control hepatocyte morphology and function in vitro. Biomaterials.

[33] Turner PA, Weeks CA, McMurphy AJ, Janorkar AV (2014). Spheroid organization kinetics of H35 rat hepatoma model cell system on elastin-like polypeptide-polyethyleneimine copolymer substrates. J. Biomed. Mater. Res. A..

[34] Turner PA, Harris LM, Purser CA, Baker RC, Janorkar AV (2014). A surface-tethered spheroid model for functional evaluation of 3T3-L1 adipocytes. Biotechnol. Bioeng..

[35] Yi A, Sim D, Lee YJ, Sarangthem V, Park RW (2020). Development of elastin-like polypeptide for targeted specific gene delivery in vivo. J. Nanobiotechnol..

[36] Kim JD, Jung YJ, Woo CH, Choi YC, Choi JS, Cho YW (2017). Thermo-responsive human α-elastin self-assembled nanoparticles for protein delivery. Colloids Surf. B Biointerfaces..

[37] Aluri SR, Shi P, Gustafson JA (2014). A hybrid protein-polymer nanoworm potentiates apoptosis better than a monoclonal antibody. ACS Nano.

[38] Cho S, Dong S, Parent KN, Chen M (2016). Immune-tolerant elastin-like polypeptides (iTEPs) and their application as CTL vaccine carriers. J. Drug Target..

[39] Huang K, Zhu L, Wang Y, Mo R, Hua Z (2017). Targeted delivery and release of doxorubicin using a pH-responsive and self-assembling copolymer. J. Mater. Chem. B..

[40] MacKay JA, Chen M, McDaniel JR (2009). Self-assembling chimeric polypeptidedoxorubicin conjugate nanoparticles that abolish tumours after a single injection. Nat. Mater..

[41] Vassilvitskii, S. & Arthur D. "k-means++: The advantages of careful seeding. In *Proceedings of the Eighteenth Annual ACM-SIAM Symposium on Discrete Algorithms* (2006).

[42] 2.3. Clustering—scikit-learn 0.24.1 documentation https://scikit-learn.org/stable/modules/clustering.html (Accessed 25 Jan 2021).

[43] Trabbic-Carlson K, Meyer DE, Liu L, Piervincenzi R, Nath N, LaBean T, Chilkoti A (2004). Effect of protein fusion on the transition temperature of an environmentally responsive elastin-like polypeptide: A role for surface hydrophobicity?. Protein Eng. Des. Sel..

[44] Weeks CA, Aden B, Kilbey SM, Janorkar AV (2016). Synthesis and characterization of an array of elastin-like polypeptide–polyelectrolyte conjugates with varying chemistries and amine content for biomedical applications. ACS Biomater. Sci. Eng..

[45] Sun C, Tang T, Uludağ H, Cuervo JE (2011). Molecular dynamics simulations of DNA/PEI complexes: Effect of PEI branching and protonation state. Biophys. J..

[46] Kobayashi S, Shirasaka H, Suh K-d, Uyama H (1990). Viscosity Behaviors and gel properties of linear and branched polyethyleneimines: Effects of micro-structures. Polym. J..

