# Supplementary material for: Machine learning to determine optimal conditions for controlling the size of elastin-based particles
Source: Sci Rep. 2021 Mar 18;11:6343. doi: 10.1038/s41598-021-85601-y (PMC7973436; doi:10.1038/s41598-021-85601-y)
Supplement: Supplementary file 1 — Supplementary Information 1. [file 41598_2021_85601_MOESM1_ESM.pdf]

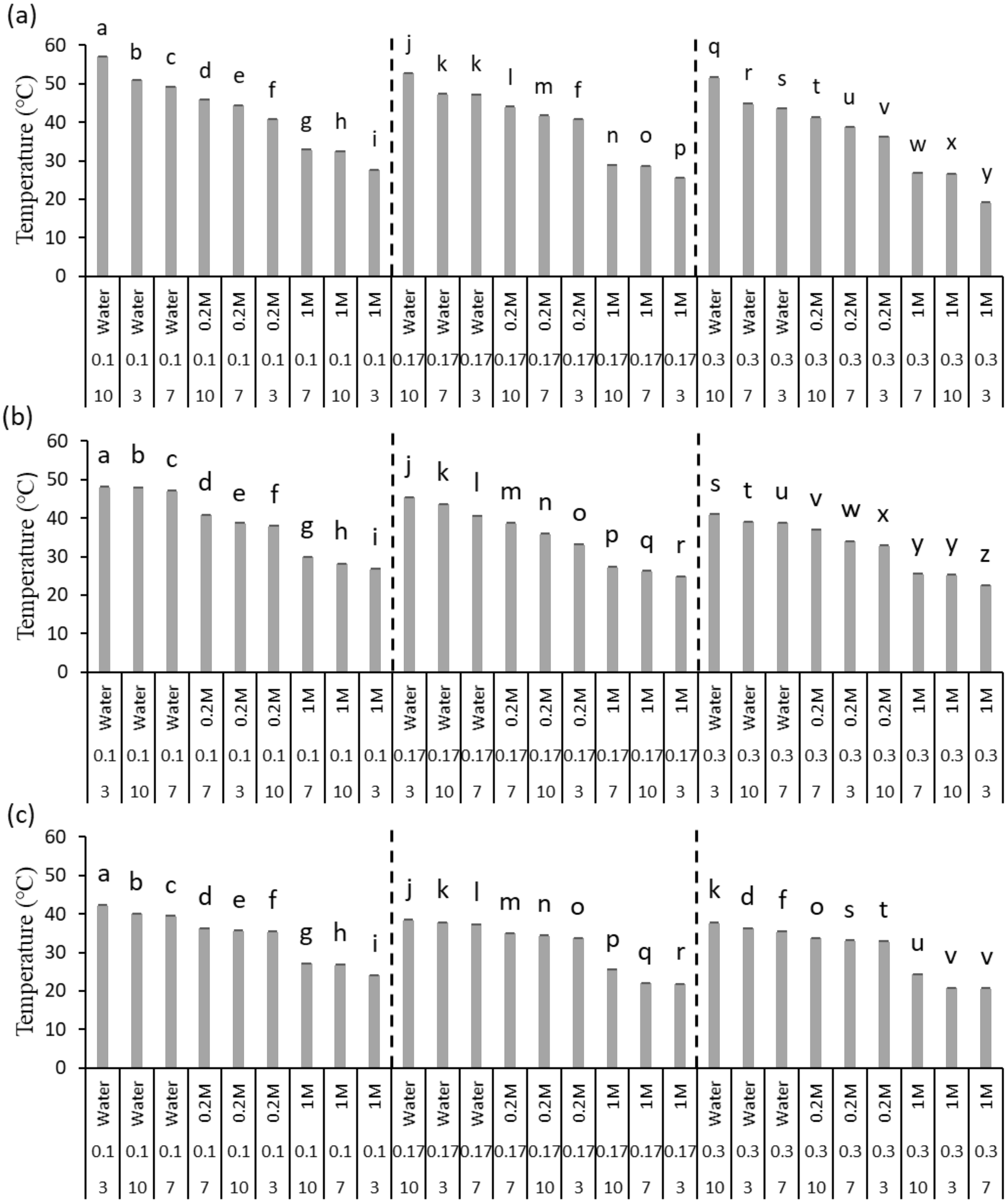

Supplemental Figure S1. Transition temperature statistical grouping for (a) ELP, (b) ELP/ELP-PEI800, and (c) ELP/ELP-PEI10K. Similar letters indicate no statistical difference between samples.

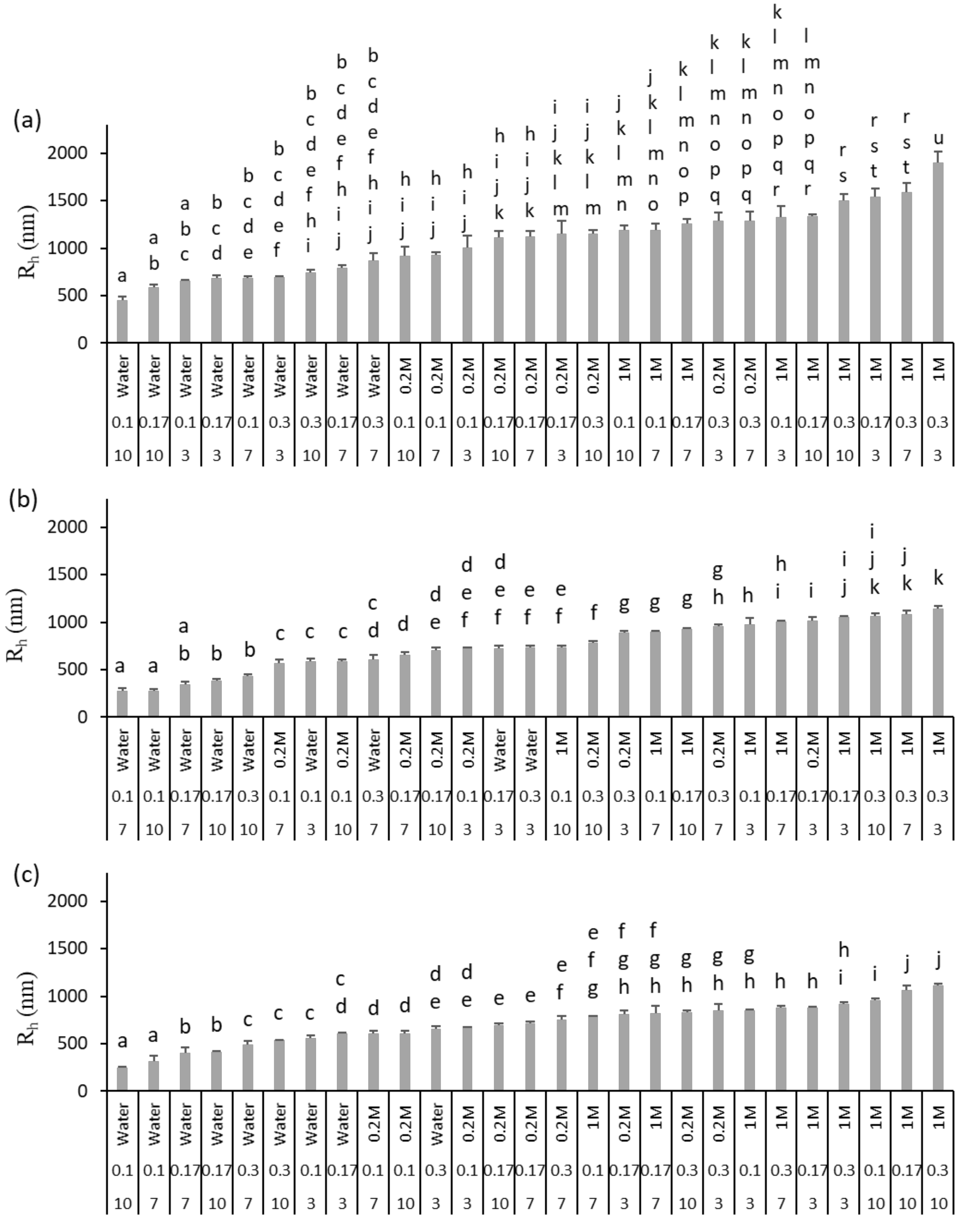

Supplemental Figure S2. Hydrodynamic radius statistical grouping for (a) ELP, (b) ELP/ELP-PEI800, and (c) ELP/ELP-PEI10K. Similar letters indicate no statistical difference between samples. R_h values incrementally increase in size which leads to overlap in statistical grouping. Thus, one sample may belong to multiple groups.
