# Supplementary material for: Machine learning to determine optimal conditions for controlling the size of elastin-based particles
Source: Sci Rep. 2021 Mar 18;11:6343. doi: 10.1038/s41598-021-85601-y (PMC7973436; doi:10.1038/s41598-021-85601-y)
Supplement: Supplementary file 2 — Supplementary Information 2. [file 41598_2021_85601_MOESM2_ESM.pdf]

In [1]:
```
import pandas as pd
import sklearn as sk
import matplotlib.pyplot as plt
import os
from sklearn import cluster
from scipy.spatial import distance
import sklearn.datasets
from sklearn.preprocessing import StandardScaler
```

In [2]:
```
Data = pd.read_csv('ELP Particles Final.csv')
```

In [3]:
```
#Modified from: https://scikit-learn.org/stable/auto_examples/cluster/plot_kme
ans_silhouette_analysis.html
from sklearn.datasets import make_blobs
from sklearn.cluster import KMeans
from sklearn.metrics import silhouette_samples, silhouette_score
import matplotlib.cm as cm
import numpy as np

print(__doc__)


#MaxRad
y = Data.iloc[:,-2].values
#Ton
X = Data.iloc[:,-1].values.reshape(-1,1)

range_n_clusters = [2, 3, 4, 5, 6]

for n_clusters in range_n_clusters:
    # Create a subplot with 1 row and 2 columns
    fig, (ax1, ax2) = plt.subplots(1, 2)
    fig.set_size_inches(18, 7)

    # The 1st subplot is the silhouette plot
    # The silhouette coefficient can range from -1, 1 but in this example all
    # lie within [-0.1, 1]
    ax1.set_xlim([-0.1, 1])
    # The (n_clusters+1)*10 is for inserting blank space between silhouette
    # plots of individual clusters, to demarcate them clearly.
    ax1.set_ylim([0, len(X) + (n_clusters + 1) * 10])

    # Initialize the clusterer with n_clusters value and a random generator
    # seed of 10 for reproducibility.
    clusterer = KMeans(n_clusters=n_clusters, random_state=10)
    cluster_labels = clusterer.fit_predict(X)

    # The silhouette_score gives the average value for all the samples.
    # This gives a perspective into the density and separation of the formed
    # clusters
    silhouette_avg = silhouette_score(X, cluster_labels)
    print("For number of clusters (k) =", n_clusters,
          "The silhouette score is :", silhouette_avg)

    # Compute the silhouette scores for each sample
    sample_silhouette_values = silhouette_samples(X, cluster_labels)

    y_lower = 10
    for i in range(n_clusters):
        # Aggregate the silhouette scores for samples belonging to
        # cluster i, and sort them
        ith_cluster_silhouette_values = \
            sample_silhouette_values[cluster_labels == i]

        ith_cluster_silhouette_values.sort()

        size_cluster_i = ith_cluster_silhouette_values.shape[0]
```

```
        y_upper = y_lower + size_cluster_i

        color = cm.nipy_spectral(float(i) / n_clusters)
        ax1.fill_betweenx(np.arange(y_lower, y_upper),
                          0, ith_cluster_silhouette_values,
                          facecolor=color, edgecolor=color, alpha=0.7)

        # Label the silhouette plots with their cluster numbers at the middle
        ax1.text(-0.05, y_lower + 0.5 * size_cluster_i, str(i))

        # Compute the new y_lower for next plot
        y_lower = y_upper + 10  # 10 for the 0 samples

    ax1.set_xlabel("Silhouette Coefficient Values")
    ax1.set_ylabel("Cluster Label")

    # The vertical line for average silhouette score of all the values
    ax1.axvline(x=silhouette_avg, color="red", linestyle="--")

    ax1.set_yticks([])  # Clear the yaxis labels / ticks
    ax1.set_xticks([-0.1, 0, 0.2, 0.4, 0.6, 0.8, 1])

     # 2nd Plot showing the actual clusters formed
    colors = cm.nipy_spectral(cluster_labels.astype(float) / n_clusters)
    ax2.scatter(X,y , marker='o', s=30, lw=0, alpha=0.7,
                c=colors, edgecolor='k')



    ax2.set_title("Clustered Data.")
    ax2.set_xlabel("1/T")
    ax2.set_ylabel("ln(Max Rad)")
    ax2.set_xticks([.015,.02,.025,.03,.035,.04,.045,.05])

    plt.suptitle(("Silhouette analysis for KMeans clustering"
                  " with number of clusters (k) = %d" % n_clusters),
                 fontsize=14, fontweight='bold')

plt.show()
```

```
Automatically created module for IPython interactive environment
For number of clusters (k) = 2 The silhouette score is : 0.5732718494832034
For number of clusters (k) = 3 The silhouette score is : 0.624400144289033
For number of clusters (k) = 4 The silhouette score is : 0.5775497713292058
For number of clusters (k) = 5 The silhouette score is : 0.5974404782535832
For number of clusters (k) = 6 The silhouette score is : 0.5813695357442924
```

Silhouette analysis for KMeans clustering with number of clusters (k) = 2

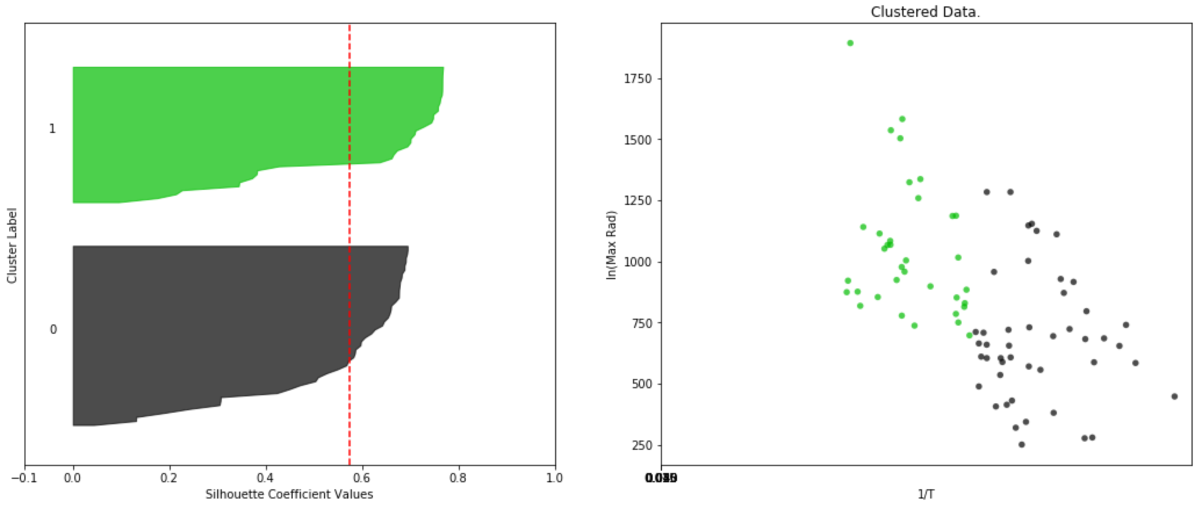

Silhouette analysis for KMeans clustering with number of clusters (k) = 3

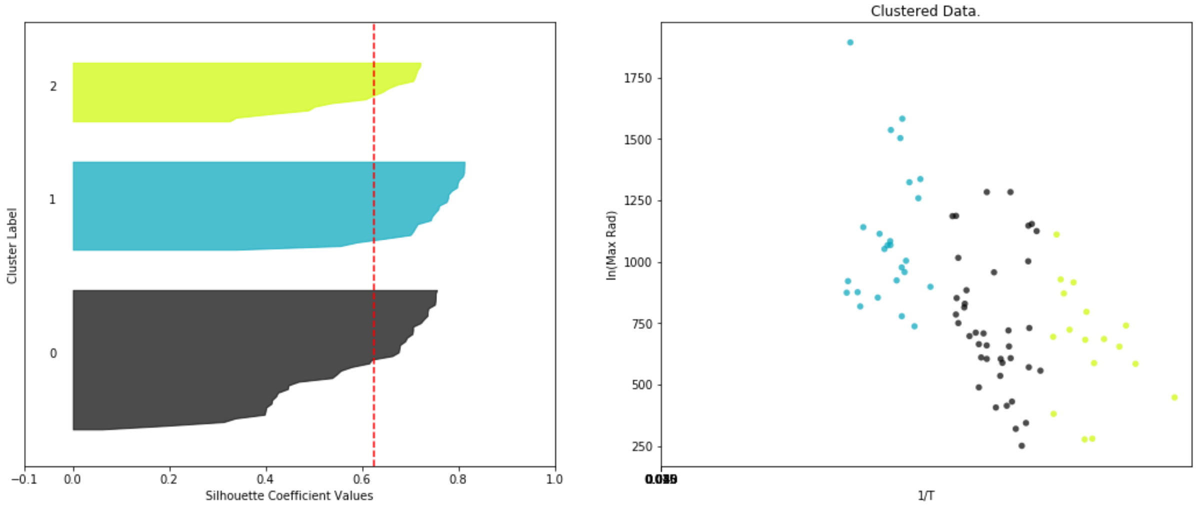

Silhouette analysis for KMeans clustering with number of clusters (k) = 4

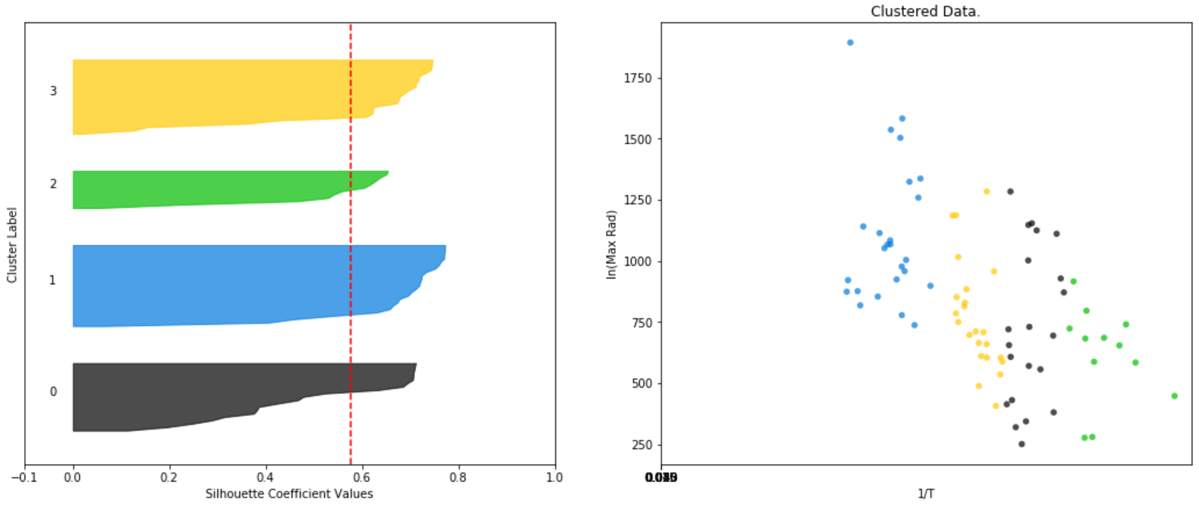

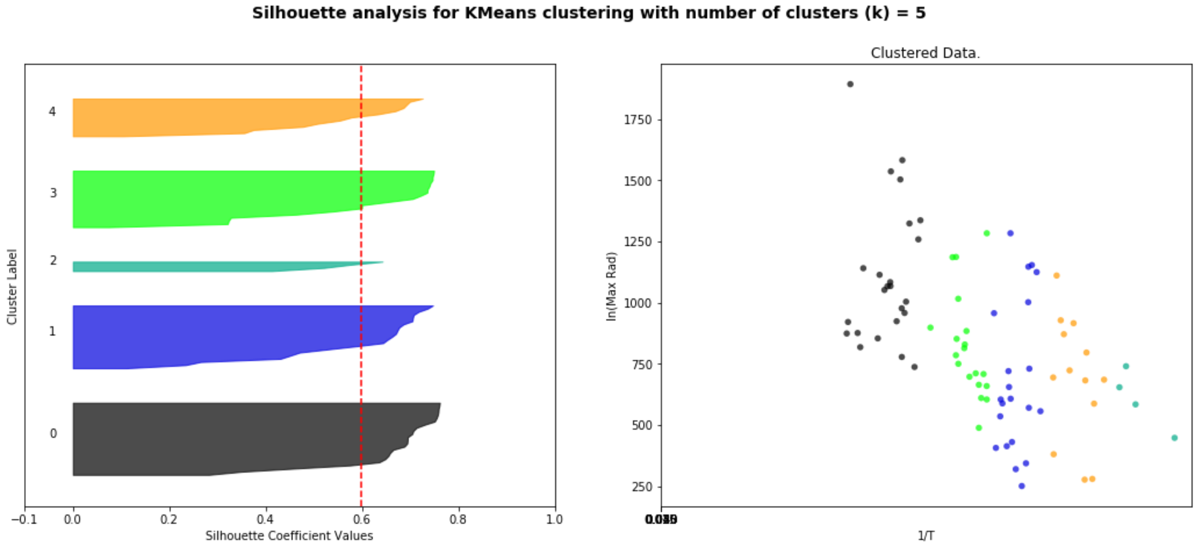
Silhouette analysis for KMeans clustering with number of clusters (k) = 5
Cluster Label
Silhouette Coefficient Values
Clustered Data.
ln(Max Rad)
1/T

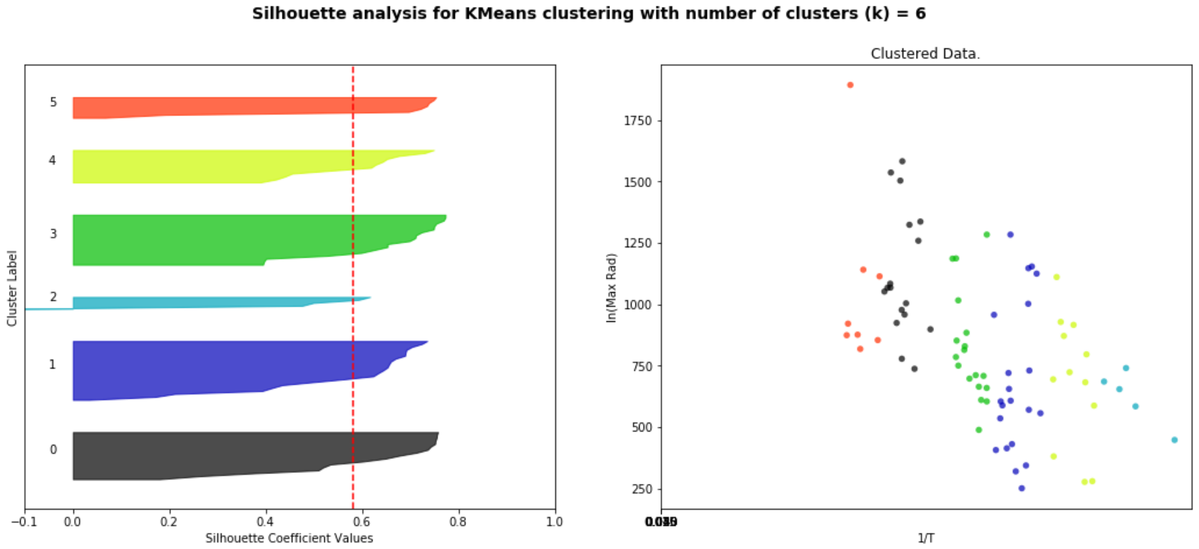
Silhouette analysis for KMeans clustering with number of clusters (k) = 6
Cluster Label
Silhouette Coefficient Values
Clustered Data.
ln(Max Rad)
1/T

In [ ]:
